# Unhealthy Breath

**Published:** 1888-05

**Authors:** Alfred H. Tester


ARTICLE III.

### UNHEALTHY BREATH.

BY ALFRED H. TESTER, L. D. S. ENG., D. M. D. HARV.

[Read before the Brighton Dental Society, December 10t , 1887.]

The subject which I have chosen to speak to you about this evening is not, I know, an inviting one. It is shunned by most dental writers, and articles treating of it in our journals are very rare.

The question naturally arises in our minds, What is the reason of this almost total neglect of this very important topic, which, if not absolutely belonging to our specialty, is one so intimately connected with it, that it should interest every member of our profession?

I cannot but think that the neglect is occasioned by want of knowledge of its primary causes, and a lack of general knowledge of the relations of all the organs (the one to the other) that work together for the sustenance and maintenance of the life and health of our bodies.

I am sure the existence of it is pretty patent. Does it not intrude on us everywhere—in the street, railway carriage, as well as our homes? Are we not annoyed at every turn—at the place of amusement, at the church and the lecture? I am sorry to say that we are obliged to turn away in disgust ofttimes when conversing with the intelligent, from the foul odour which comes from their breath. Are we not sorry within ourselves, and is not our pity greater than our disgust for those unfortunately so afflicted?

The love of purity and sweetness is inherent in our natures; a sweet breath, then, becomes a fortune and a blessing. But, as a class, who does it annoy more than the dentist, who, hour after hour and day after day has to earn his living by bending over the "gate-way of life," the breathing-places of human beings, taking into his central vitality the putrid odours which emanate from some of our patients. But, alas! for the weakness of humanity. "They that live in glass houses should never throw stones," and we who criticise are not exempt from the plague, and I am sure we should look to ourselves before we preach to our patients.

Doubtless, the causes of unhealthy breath are numerous. Temporarily, it may be produced by any nauseous or ill-flavored substance, either chewed or used as food, such as garlic, onions, tobacco, &c., or by bad odoured drinks; by simple opposition to the teeth and mouth of remnants of food getting foul; and by diseases of the teeth and gums, which could at all times be averted by regular habits of cleanliness and judicious treatment—for instance, by washing the mouth and brushing the teeth constantly, and especially the last thing at night, and having all cavities filled, unhealthy roots extracted, and diseases of the gums cured.

Other causes are found in those suffering from dyspepsia and constitutional derangements of the system.

There are also a great many persons affiicted with this unhealthy tendency, who have no idea of its cause through any act of their own, and are as innocent of the existing trouble as a newly-born babe.

We find that we must seek for the cause of the odour in emanations of the glands, placed along the mucous iract, from its commencement to its close, and, of course, more particularly those of the muuth, throat and nasal passages.

We may also seek for it in the air from the lungs, as an emanation from the blood itself.

There may be many other causes, but if we understand the philosophy of the cause of disease in these parts, we probably shall be able to understand it in other parts of the system.

On examining the condition of health, we find that the system may, in its relation to food, be divided into three separate functions :--First, the organs of *reception*, viz., the mouth, teeth, and glands, salivary and tonsilary, &c., preparatory to entering the second—the stomach—portion of the alimentary canal with pancreas, liver, and surrounding viscera, which may be termed the organs of *assimilation*; and third, the remainder of the intestinal tract, kidneys, &c., called the organs of *excretion*.

The functions of all these parts are strictly marked, and never, under the conditions of absolute health, does one organ interfere with the other, but in an overworked, or diseased condition of an organ some other one may assist it in its duties, as the skin, which under ordinary conditions secretes a lubricating fluid for the protection of its surface and adjusting the heat of the body, may carry off through its pores the emanations that more properly belong to the kidneys, bladder, or intestines.

The *"interchange of function,"* well known to physiologists, assists us to understand our subject, for, as a rule, all the impurities of the system should be removed by the organs of excretion when they are in a state of health.

Let any of these three organs be out of order, as for example, the motions of the rejecting canal be retarded, the accumulated secretion must find some other exit. Therefore, when there is any disagreeable odour present there must be some poisonous matter confined to cause it; matter that should be eliminated, and which is detrimental to health, and therefore the organs containing it are in a state of disease.

Weakness in the organs of assimilation causes an irritating of the surface of the stomach, which brings on an increase of activity of the blood vessels, and ends in the inflammation of the part.

An illustration of this, is the case of an overloaded stomach. Persons wno are liable to chronic bad health will have it come on under such a condition; an hour or two after a meal a tainted breath is noticed, it increases rapidly and constantly, until the stomach appears to be entirely empty. In some cases even then it may continue and increase, but if fasting is continued long enough, the unhealthy taste in the mouth and tainted breath are overcome and purity returns.

If the organs below the stomach do their duty, there will be less chance of unhealthy breath, but if not so, foul gases will be evolved by imperfect digestion and the fermentation of more food than the stomach can wrestle with.

The proper balance of diet in its vegetable, animal, and mineral constituents will, as far as diet is concerned, produce health, but it is necessary that all the surplus that is not retainable should be thoroughly eliminated from the system; but to do this the excretory organs must be in active working condition; they should not be coaxed into working order by the aid of aperients.

If these organs are not capable of this duty, and cannot fulfil it completely, some portion of the debris must remain in the system, and as it is a poison, it cannot be stored in any one organ without causing danger of a fatal sickness.

Nature, by the same mode of interchange of function, distributes the foreign matter throughout all organs that possess any excretory powers. She loads the blood with it, she lines the air passages of the lungs with it, and also forces it out of the skin.

Wherever nature can oxidise the substances, it is done; but where it accumulates in large quantities, it attacks the delicate tissue of the lungs by a kind of spontaneous combustion.

I think it will be found that certain habits will bring on and continue unhealthy breath, and one of the most common causes is found in persons suffering with constricted, irregular and costive habits of the bowels, and that for its cure, particular attention to regulating this portion of the system is absolutely necessary.

Overloaded and dyspeptic conditions of the stomach are the next causes of unhealthy breath. Let any one who has a tendency to inflamed condition of the mucous coat of the mouth, throat, and surrounding parts, eat an over-full meal of even the simplest substances, under conditions that do not favour the digestion of it and it will bring on the trouble. A hearty meal just before bedtime will probably produce it during the night, so that on awakening in the morning the mouth will be noticeably foul and dry. So also will the tendency be kept up by too frequent meals.

It would be better that we had an interval of six hours between meals, so that the stomach should have time to rest, and the food time to digest.

Articles of food taking five hours to digest cannot be done in three, and no food should be mixed with partly digested material. This, I am sure, is one of the most fruitful causes of inflamed mucous membrane and unhealthy breath.

Spices and condiments, as a rule, should be avoided with food, as they form compounds with animal and vegetable substances, thereby retarding and preventing decay.

A very good rule is: that all articles of food easily decomposed by warmth and moisture out of the human stomach are easily digested in the stomach, and all articles of food which are slow to decompose under such conditions are slow of digestion, the ratio of digestive process being proportionate to the process of decomposition.

Large quantities of fluids are irritants to some people, by floating food and preventing digestion, and if the simple ones as milk and water are, what can quantities of rum, gin and other spirits cause but an irritation to the mucous membrane, a bad taste in the mouth, and unhealthy breath.

I believe we may get complications from unhealthy breath in the form of phlegm and catarrhal troubles.

Another cause of unhealthy breath is due from want of proper mastication, which is very prevalent in our children, who bolt their food, half chewed. Rule:—If you want your children healthy, teach them to chew their food fine and to eat slowly.

Everything that tends to disturb the digestion, either from internal or external causes, will prevent the cure of this disease. Want of exercise, too much worry and mental strain, and too much sensuality.

To persons who are subject to the direct action of mercury, lead, phosphorus, and some of the dyeing processes, have the mucous membrane and bones of the mouth affected by these metals, and are the subjects of very fœtid odours, and complications of blood poisoning soon follow.

I think that one of the greatest causes of this misfortune which attacks some of the members of our profession, as well as other people, is that brought on by indigestion, caused by imperfect mastication in the hurry of professional life, bringing on an irritative dyspepsia, acidity and flatulence.

To those to whom this disease comes as an infliction, we must tender our regret, but those whose mouths are nasty by their own filth, uncleanliness and dirty habits, we must withhold our symphthy.

We are members of a profession that teaches cleanly habits, and to make our teachings effectual, we must be cleanly ourselves. Preaching is no good without the practice. One cannot conceive how any dentist can approach his patients with a befoulment of onions and tobacco in his mouth; but disagreeable as this is to most patients, it is quite sweet to those whose clothes as well as their mouths are saturated with the stale odours of second-hand cigars and pipes that some of my friends are in the habit of using, being quite innocent of their faults.

As regards the treatment of this subject, any dentist who discovers that he has a tainted breath should always when working over a patient close his mouth tightly and breath through his nose. Have at hand some disinfectant mouth wash, and keep the mouth frequently rinsed at intervals.

Another palliative is to keep the mouth wet with fresh saliva by chewing some inodorous substance, as a piece of paper or gum elastic. Do not use cachous, as the remedy is often as bad as the disease.

A very good antidote for offensive breath is the concentrated solution of chloride of soda, six to ten drops in a wine glass of water, to be used after the toilet is completed in the morning. In cases where the odour from carious teeth is combined with that from the stomach, a teaspoonful of the solution in a tumbler of water will act as a charm, a drop of wintergreen being added to make it pleasant.

Patients suffering from this unhealthiness should, after having been advised to use local remedies, study the necessity of taking more active exercise, together with abstinence, inhale and exhale the pure air, oxidize the food and blood that will kill the odours.

In concluding my paper, which I fear may have been dull and uninteresting to some, I emphasize my wish that the members of the dental profession should give more attention to this most important subject.—*Dental Record.*

